# Development and Validation of a UHPLC–MS/MS Method for the Quantification of a Novel PYGB Inhibitor in Plasma: Application to Pharmacokinetic Studies

**DOI:** 10.3390/molecules28196995

**Published:** 2023-10-09

**Authors:** Sumei Xu, Shuai Li, Zhiwei Yan, Youde Wang, Liying Zhang

**Affiliations:** Laboratory of Traditional Chinese Medicine Research and Development of Hebei Province, Institute of Traditional Chinese Medicine, Chengde Medical University, Chengde 067000, China; 15036252320@163.com (S.X.); cmuyhls@163.com (S.L.); cdyanzhiwei@163.com (Z.Y.);

**Keywords:** PYGB inhibitor, UHPLC–MS/MS, pharmacokinetics, brain tissue distribution, ischemic brain injury

## Abstract

In previous studies, we reported compound **1** (5-chloro-*N*-(4-oxo-2,2-dipropyl-3,4-dihydro-2*H*-benzo[*e*][1,3]oxazin-6-yl)-1*H*-indole-2-carboxamide) as a novel PYGB inhibitor, and found that it had better anti-ischemic brain injury activity. In this study, we established and validated a novel UHPLC–MS/MS method for the quantitative determination of compound **1** in plasma, then applied the method to study the pharmacokinetic parameters and brain tissue distribution of compound **1** in SD (Sprague—Dawley) rats after intravenous administration. The experimental results showed that the method met the validation requirements set by the US FDA in terms of linearity, accuracy, precision, and stability. The validated method was then used for pharmacokinetic studies in rat plasma, and it was found that compound **1** exhibited linear pharmacokinetic characteristics when administered in the dose range of 0.8–3.2 mg/kg. Finally, we also conducted a brief preliminary investigation of the brain tissue distribution of compound **1** in rats after injection and found that the brain tissue concentrations at 0.25 h and 2 h of administration were 440 ± 19.1 ng/kg and 111 ± 23.9 ng/kg, respectively. Additionally, the C_Brain_/C_Plasma_ ratio was 0.112 ± 0.0185 and 0.112 ± 0.0292, respectively. These results indicated that compound **1** was able to cross the blood–brain barrier. This study provides important support for the application of compound **1** in ischemic brain injury diseases.

## 1. Introduction

Ischemic brain injury is a common disease that seriously threatens human life and health [1,2,3]. It is characterized by a high fatality rate, high disability rate and high recurrence rate [4,5,6,7,8]. However, there is currently no effective clinical treatment strategy [9,10,11]. In addition, many patients have lost their lives because effective treatment is required within a short period of time. At present, it has been found that the overactivation of brain glycogen phosphorylase (PYGB) was one of the critical factors in brain tissue injury during ischemic stroke [12,13,14,15,16,17,18]. Therefore, inhibition of PYGB overactivation is expected to be a new approach to prevent and treat ischemic stroke. In addition, the PYGB inhibitor has been recognized as a kind of prospective drug for treating ischemic brain injury [14,15,19,20], and as a potential novel anti-ischemic stroke drug has a favorable prospect for research and development.

**Figure 1:**
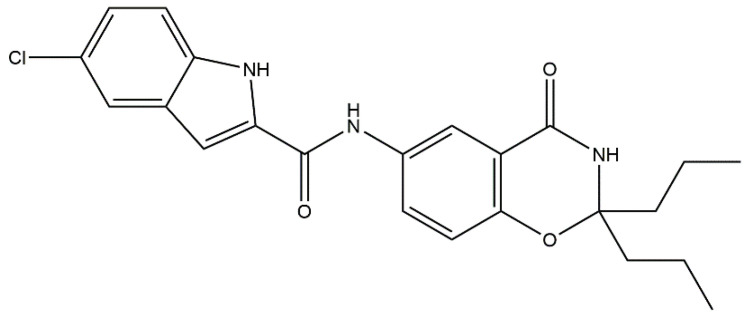
The structure of compound **1** (C_23_H_24_ClN_3_O_3_, exact mass: 425.15 g/mol). (5-chloro-*N*-(4-oxo-2,2-dipropyl-3,4-dihydro-2*H*-benzo[*e*][1,3]oxazin-6-yl)-1*H*-indole-2-carboxamide).

In previous studies, we reported compound **1** as a novel PYGB inhibitor (Figure 1). The IC_50_ of PYGB inhibition was 90.27 nM, and it showed a better protective effect against ischemic brain injury [14,15,21]. Although compound **1** had been studied and reported in relation to ischemic brain injury by us, its pharmacokinetic parameters have not been analyzed. Pharmacokinetic analysis is an indispensable link in the process of drug research and development, which can provide an important scientific basis for drug research and development and clinical application [22,23]. In addition, intravenous administration has become the current recommended treatment for ischemic disease [24,25,26], so we analyzed the pharmacokinetic parameters of compound **1** in SD rats in this way.

In this study, we successfully developed a new UHPLC–MS/MS assay method to analyze the pharmacokinetic parameters of compound **1** in plasma after intravenous injection. The distribution of compound **1** in brain tissue was preliminarily investigated. This study provides new data support to further explore the mechanism of action of compound **1**.

## 2. Results and Discussion

### 2.1. Method Development

In order to optimize the intensity of the mass spectral response for compound **1** and psn357 (IS) to obtain the desired sensitivity, 1 mg/mL of standard working solution was injected into the mass spectrometer source at a flow rate of 0.01 mL/min, and the intensity of the response was determined in the positive and negative ion modes. The results showed that both analytes and internal standards responded better in the positive mode.

For the purpose of achieving the best possible separation of the two analytes, the column and mobile phase compositions were optimized even further. In this experiment, we tried two columns including the ACQUITY UPLC BEH C18 column and the Kinetex 2.6μ XB-C18. It was found that compound **1** and IS were retained better on the ACQUITY UPLC BEH C18 column (2.1 × 50 mm, 1.7 µm). Similarly, different mobile phase compositions including water–methanol and water–acetonitrile were investigated to obtain the desired peak shapes and better separation of the analytes. It turned out that if a solvent mixture of methanol and water (4:1, *v*/*v*) was chosen as the mobile phase, no interfering peaks were observed at the retention times of 1.60 min (compound **1**) and 0.80 min (IS). In order to shorten the analysis time and increase the efficiency of the experiment, a 5-min isocratic elution over the column was optimized. The column temperature of 30 °C, flow rate of 0.3 mL/min and injection volume of 2 µL were also optimized. In the end, the separation of analytes was achieved with good peak shapes and high mass response to meet the experimental requirements.

### 2.2. Method Validation

#### 2.2.1. Selectivity

**Figure 2:**
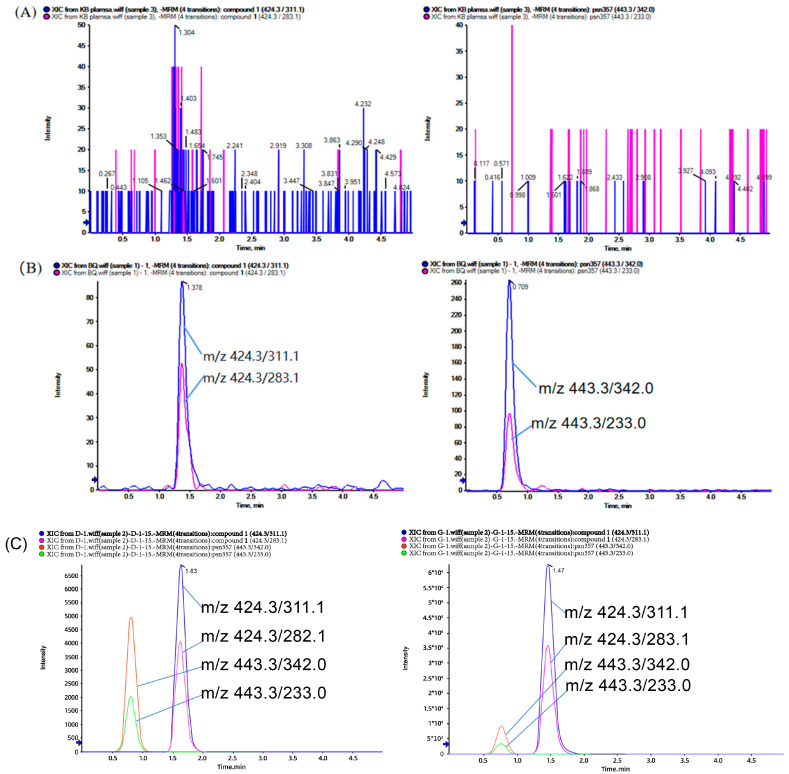
Representative MRM profiles of compound **1** and psn357 (IS) in rat plasma. Blank rat plasma sample (**A**); blank plasma sample with 1 ng/mL of compound **1** and psn357 added (**B**); and 15-min plasma sample taken from a rat receiving 0.8, 3.2 mg/kg of compound **1** (**C**).

Selectivity refers to the ability of an analytical method to differentiate and quantify analytes in the presence of other components in a plasma sample [27,28]. The mass spectra of different plasma samples injected and analyzed are shown in Figure 2, from which we can see that there is a significant difference in the content of compound **1** in the plasma of administered and unadministered rats, and we also found that the peak positions of compound **1** and psn357 are not significantly interfered with by endogenous substances. These results indicated that the method had good selectivity and met the requirements for the analysis of biological samples.

#### 2.2.2. Linearity and the Lower Limits of Quantitation

We found that the peak area was linearly correlated with the concentration of compound **1** in the range of 1.0~1000.0 ng/mL. The equation of the standard curve was Y = 0.00152X + 0.01389, where Y represented ratio of the peak area of compound **1** to the internal standard psn357, and X represented compound **1** concentration (ng/mL). The coefficient of determination was found to be 0.99830. The lower limit of quantitation was 1.0 ng/mL.

#### 2.2.3. Accuracy and Precision

After the above analysis, we also conducted accuracy and precision tests, and the results are shown in Table 1. The accuracy of compound **1** ranged from −1.05% to 3.99%. The intra-day and inter-day precisions for compound **1** ranged from 4.45% to 11.33%. The accuracy was measured using relative error (RE), and intra-day and inter-day precisions were measured using relative standard deviation (RSD).

#### 2.2.4. Matrix Effect and Extraction Recovery

The matrix effect and extraction recovery can reflect the influence of the matrix on the drug, which are validation metrics for establishing a good liquid chromatography-mass spectrometry method in pharmacokinetic studies [26,27,28,29]. Therefore, we analyzed and validated the matrix effect and extraction recovery of compound **1** using this method. The results in Table 2 showed that the matrix effect range of compound **1** was 91.07–97.69%, and the extraction recovery range was 91.55–98.35%. The matrix factor range normalized by the internal standard was further calculated to be 97–105% by using the matrix factor data for compound **1** and the internal standard in the table below. These results all showed that the method had no obvious matrix effect, and the extraction recovery was good enough, which could meet the analytical requirements of compound **1**.

#### 2.2.5. Stability

A better drug stability is the basis for accurate sample analysis [30,31,32], so we analyzed the stability of compound **1** under the different conditions, and the results are shown in Table 3. The results showed that compound **1** had better stability under the four different conditions, and it could meet the requirements of the study on the metabolism of compound **1** in plasma.

#### 2.2.6. Dilution Integrity

The reliability of the dilutions was assessed based on the precision and accuracy of results by analyzing samples of 10,000 ng/mL of compound **1** diluted 10-fold, 20-fold and 100-fold. As shown in Table 4, the precision and accuracy of the diluted samples were within ±15% of each other, which met the requirements for the analysis of biological samples.

### 2.3. Pharmacokinetic Studies

**Figure 3:**
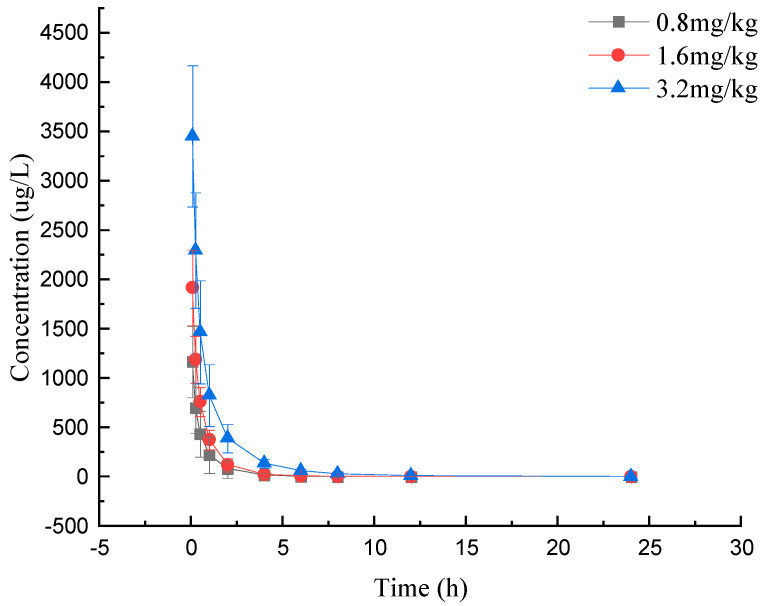
Mean concentration–time (c-t) profiles for compound **1** administered at different doses.

The above results confirmed that this method was in line with the UHPLC–MS/MS assay in the FDA validation guidelines, and we successfully applied the method to the quantitative analysis of compound **1** in rat plasma. Compound **1** was administered intravenously to rats at low, medium and high doses (0.8, 1.6 and 3.2 mg/kg), and its metabolism in plasma was analyzed, with the results shown in Figure 3 and Table 5.

We found the concentration–time relationship of compound **1** in rat plasma after three doses, Figure 3. The results showed that the concentration of compound **1** in rat plasma decreased rapidly, indicating that compound **1** may be rapidly distributed into tissues and organs or metabolized and excreted after injection into rat plasma.

By analyzing the results in Table 5, we found that in the three groups of pharmacokinetic parameters, the values of AUC_0–t_, AUC_0–∞_ and C_0_ increased in a concentration-dependent manner with the increase in concentration. Additionally, there was no significant difference of CL and T_1/2_. These results demonstrated a linear pharmacokinetic profile of compound **1** in rat plasma when administered in the range of 0.8–3.2 mg/kg. In addition, from the table below, the pharmacokinetic parameter, Vd, after the administration of low, medium and high doses to rats was 6.43 ± 3.48, 7.51 ± 6.15 and 4.14 ± 1.23 L/kg, respectively, which was much larger than the total blood volume of the rats, which indicated that the drug might have entered into the organs and tissues or been metabolized after injection in the rats. This laid a foundation for the following study on the blood–brain barrier permeability of the drug.

### 2.4. The Distribution of Compound ***1*** in Brain Tissue

From the above results, we found that compound **1** could quickly enter tissues or be metabolized in rats after intravenous injection. Meanwhile, the distribution and elimination of compound **1** in vivo was consistent with a linear pharmacokinetic profile over the dose range of 0.8–3.2 mg/kg. We have previously reported that compound **1** had a better effect on cerebral ischemia [14,15]. Therefore, we preliminarily explored the distribution of compound **1** in brain tissue after intravenous injection at a dose of 3.2 mg/kg.

The results in Table 6 show the concentration of compound **1** in rat plasma and brain tissue at different time points after intravenous administration at a dose of 3.2 mg/kg. We found that the concentration of compound **1** in brain tissue was 440 ± 19.10 ng/g, 111 ± 23.90 ng/g and 4.17 ± 7.22 ng/g, respectively, 0.25 h, 2 h and 8 h after intravenous administration.

The C_Brain_/C_Plasma_ ratio results of compound **1** in rats at different time points are shown in Table 7. The C_Brain_/C_Plasma_ ratios of compound **1** at different time points in rats were 0.11 ± 0.019, 0.11 ± 0.029 and 0.09 ± 0.148, respectively. The results above indicated that compound **1** could quickly cross the blood–brain barrier, and the discovery of compound **1** could provide a new possibility for the treatment of cerebral ischemia diseases.

At the same time, the pharmacokinetic parameters of compound **1** in plasma and brain tissue were analyzed (Table 8). We found that compared with plasma pharmacokinetic parameters, the C_max_ and AUC_0–∞_ of the pharmacokinetic parameters in brain tissue were significantly lower, and the T_1/2_ and MRT were not significantly different, indicating that compound **1** had similar metabolic characteristics in plasma and brain tissue. However, due to the existence of the blood–brain barrier, the concentration of compound **1** entering the brain tissue was significantly lower than that in the plasma, which also indicated that the blood–brain barrier controlled the entry of drugs into the brain tissue to better maintain the stability of the brain environment. These results provide better theoretical support for the further study of the distribution of compound **1** in brain tissue and its pharmacodynamics.

## 3. Materials and Methods

### 3.1. Chemicals and Reagents

Compound **1** was a novel GP inhibitor that we previously reported (purity ≥ 97%) [14,15], and psn357 was used as an internal standard manufactured by the Chemical Synthesis Laboratory of Traditional Chinese Medicine, Chengde Medical College. PBS was purchased from Beijing Huarui Guangnian Culture Development Co., Ltd. (Beijing, China), and pure water was purchased from Watson’s Distilled Water Co., Ltd. (Beijing, China). DMSO, methanol, isopropanol, acetonitrile, formic acid and other chemicals were of high-performance liquid chromatography (HPLC) grade and were purchased from official sources.

### 3.2. Preparation of Stock Solutions

A total of 1.00 mg of compound **1** control was weighed precisely and prepared in 1 mL of DMSO solution for a stock solution concentration of 1 mg/mL, which was diluted stepwise with a solvent mixture of methanol and water (4:1, *v*/*v*) to a series of standards at concentrations of 10, 50, 100, 500, 1000, 2000, 5000, 10,000 ng/mL and 10, 25, 1000, 8000 ng/mL of quality control working solution. A concentration of 100 ng/mL psn357 was used as the internal standard. To prepare the standard curve and QC samples, 5 μL of standard or QC working solution was added to 50 μL of blank plasma and mixed well for protein precipitation and centrifugation. Finally, all the stock solutions were stored in the refrigerator at 4 °C.

### 3.3. Plasma Sample Pre-Treatment

50 μL of the plasma to be tested was aspirated precisely and placed in a 1.5 mL centrifuge tube. Then 5 μL of internal standard working solution and 150 μL of methanol were added sequentially to the centrifuge tube, and the tube was vortexed for 60 s and centrifuged at 6147× *g* for 10 min. Finally, 100 μL of supernatant was taken and added to the injection vial for quantitative analysis by UHPLC–MS/MS.

### 3.4. Pre-Treatment of Brain Homogenate Samples

Brain tissue homogenate samples for analysis were prepared by adding 9 volumes of homogenate solution (MeOH/15 mMPBS (1: 2, *v*/*v*)) to one volume of brain homogenate and diluting it 10-fold. Firstly, an aliquot of 40 μL of the brain homogenate sample to be tested (diluted sample), calibration standards, QC and diluted QC, a zero sample (with internal standard) and a blank sample (without internal standard) were added to the 96-well plate. Then, each sample (except blank samples) was quenched with 400 μL of IS1 (blank samples were quenched with 400 μL of acetonitrile), and the mixture was vortexed at 800 rpm for 10 min, followed by centrifugation at 3220× *g* at 4 °C for 15 min. Finally, a 50 μL aliquot of the supernatant was transferred to another clean 96-well plate and centrifuged at 3220× *g* for 15 min at 4 °C, then injected directly into the sample for UHPLC–MS/MS analysis.

### 3.5. Chromatographic and Mass Spectrometric Conditions

*Liquid chromatography conditions*. UHPLC–MS/MS analyses were performed on an Agilent 1260 ultra-high performance liquid chromatograph (Agilent Technologies Co., Ltd., Santa Clara, CA, USA) and a QTRAP 5500 triple quadrupole tandem mass spectrometer (AB Sciex Pte. Ltd., Framingham, MA, USA). An ACQUITY UPLC BEH C18 column (2.1 × 50 mm, 1.7 µm) was used at 4 °C with a flow rate of 0.3 mL/min and an injection volume of 2 µL. The analysis time was 5 min using an isocratic elution procedure of methanol and water (4:1, *v*/*v*).

**Figure 4:**
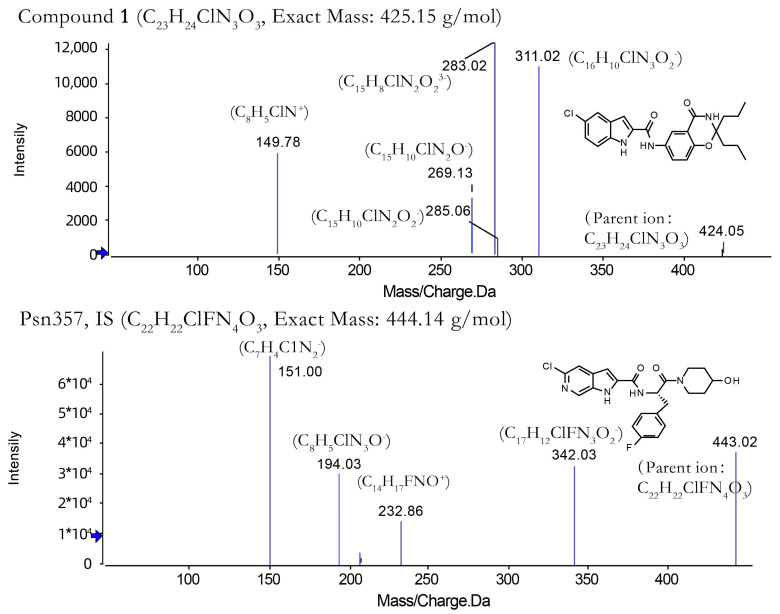
Molecular structures and TOF MS/MS of compound **1** and psn357.

*Mass spectrometric conditions*. The mass spectrometry detection ion source was a Turbo V ESI ion source with multiple reaction monitoring (MRM), negative ion detection, a source heating temperature of 550 °C, an ion spray voltage of 5.5 kV, a curtain gas, a nebulizing gas and a heating gas of 35 psi, 50 psi and 55 psi, respectively, and all gases used were nitrogen. Compound **1** and the internal standard psn357 detected ion pairs of *m*/*z* 424.300→311.100, 424.300→283.100 and *m*/*z* 443.300→342.000, 443.300→233.000 with collision energies of 35 V, 30 V, respectively. Included among these, the structure and product ion mass spectra of compound **1** and psn357 (IS) are shown below (Figure 4).

### 3.6. Method Validation

The method validation was in accordance with the US Food and Drug Administration (FDA) guidelines for the validation of biological methods [33]. The performance parameters evaluated in this experiment included selectivity, linearity and the lower limits of quantitation, accuracy and precision, matrix effects and extraction recovery, and stability.

#### 3.6.1. Selectivity

To assess the specificity of the method, chromatograms of blank rat plasma samples, blank plasma samples spiked with 1 ng/mL of compound **1**, and plasma samples collected from rats injected intravenously for 15 min at low and high doses were compared to explore the possibility of retention time interferences between analytes.

#### 3.6.2. Linearity and the Lower Limit of Quantitation

The standard curve of compound **1** in plasma was plotted by UHPLC–MS/MS analysis of plasma standard solutions (1, 5, 10, 50, 100, 200, 500, 1000 ng/mL) with the concentration of compound **1** as the horizontal coordinate (X, ng/mL) and the ratio of the peak area of compound **1** to that of the internal standard psn357 as the vertical coordinate (Y).

#### 3.6.3. Accuracy and Precision

The accuracy and precision of the method was evaluated by performing UHPLC–MS/MS analysis of quality control (QC) samples at the lower limit of quantification (LLOQ), and low, medium, and high concentrations (1, 2.5, 100, and 800 ng/mL), by preparing six samples in parallel at each concentration level, with three analytical batches spread out over three days, and calculating the concentration of each QC sample based on the standard curves accompanying each day.

#### 3.6.4. Matrix Effect and Extraction Recovery

Matrix effects and recoveries of rat plasma samples were determined by analyzing plasma from six different rats at three quality control concentration levels (2.5, 100, 800 ng/mL). Specifically, the percentage of the matrix effect was calculated by comparing the peak area response values of plasma samples spiked with compound **1** after protein extraction and water samples spiked with the compound. The recovery of compound **1** was calculated from the percentage peak area response of plasma samples with the compound added before protein extraction and after extraction. The same procedure was followed for the evaluation of matrix effects and recovery impacted on the internal standard.

#### 3.6.5. Stability

Stability was assessed under the four conditions (storage at room temperature for 12 h, ambient storage at 4 °C for 24 h, refrigerator storage at −20 °C for 10 days, and freeze-thawing at −80 °C to 25 °C for 3 cycles) using the QC working solution (2.5, 800 ng/mL), and six preparations were made in parallel for each concentration. The stability of compound **1** in rat plasma was evaluated by calculating the peak area ratio (compound **1**/IS) in plasma sample.

#### 3.6.6. Dilution Integrity

In this experiment, since compound **1** in rat plasma may exceed the upper limit of quantitation (1000 ng/mL), plasma samples with concentrations exceeding 1000 ng/mL were prepared and diluted 10-fold, 20-fold and 100-fold with blank matrix to bring them within the concentration range of the calibration curve. Then, the samples were analyzed, and the concentrations were measured in order to evaluate the reliability of the dilution of the drug in plasma.

### 3.7. Study of the Pharmacokinetics

The animal experiments were approved by the Experimental Animal Welfare Ethics Committee of Chengde Medical College (Animal Ethics Committee Approval No. CDMULAC-20210409-003). Healthy SD rats (female and male), weighing about (260 ± 10) g, were purchased from Beijing Huafukang Bio-technology Co., Ltd. (Production License No: SCXK (Beijing, China) 2019-0008, Certificate of Conformity No: No. 110322231100616953). The animals were housed in the observation room of the barrier animal house (temperature: 20~26 °C, relative humidity: 40%~70%, 12 h light/dark cycle) with free access to water and food and cleaned and disinfected daily. The animals were not allowed to eat but to drink freely 12 h before the experiment. 

In the plasma pharmacokinetic study, 30 healthy SD rats (half male and half female) were selected and randomly divided into 3 groups of 10 rats each. Three groups of SD rats were injected with a single dose of compound **1** (0.8 mg/kg, 1.6 mg/kg, 3.2 mg/kg) by tail vein, respectively. At 0.084, 0.25, 1, 2, 4, 6, 8, 12, 24 h after administration, 0.5 mL of blood was collected from the fundus venosus in heparin-anticoagulated tubes, and the plasma was separated by centrifugation at 6149× *g* for 10 min, and then placed in 1.5 mL Eppendorf tubes and stored in the refrigerator at −80 °C for measurement.

In the preliminary brain tissue distribution study, 18 healthy male SD rats were injected with 3.2 mg/kg of compound **1** intravenously into the tail, and the animals were euthanized by CO_2_ inhalation at 0.25, 2 and 8 h after the administration of compound **1**, respectively. Blood and brain tissue were then collected separately from each animal. Brain tissue was homogenized on wet ice using homogenizing buffer (MeOH/15 mM PBS = 1/2) at a ratio of 1:9 (1 g tissue to 9 mL buffer). The tissue homogenate was divided into two parts, 0.8 mL was used for analysis and the rest was discarded.

### 3.8. Data Processing

The data was processed using Origin 2021 to plot the drug concentration–time curve in plasma, and DAS 2.0 software was used to calculate the pharmacokinetic parameters by the non-atrial model statistical moment method, and the results of the experiments were expressed as mean ± standard deviation. Differences between groups were analyzed using SPSS 20.0, with *p* < 0.05 indicating statistical significance.

## 4. Conclusions

In the study, we developed and validated a novel UHPLC–MS/MS quantitative analysis method, and successfully applied it to the quantitative determination of compound **1** in plasma. This method had excellent specificity, precision, recovery and stability. At the same time, as a potential drug for the treatment of cerebral ischemia, we also conducted a preliminary analysis of the distribution of compound **1** in the brain tissue after intravenous administration. Compound **1** was found to be able to cross the blood–brain barrier and had similar metabolic characteristics in brain tissue and plasma.

In conclusion, this study provides a reliable pharmacokinetic reference for compound **1** and provides valuable information for the development and clinical application of compound **1**.

## Figures and Tables

**Table 1:** Precision and accuracy of compound **1** in plasma within and between batches (mean ± SD, *n* = 6).

Conc. (ng/mL)	Intra-Day			Inter-Day
	Mean ± SD	RSD (%)	RE (%)	RSD (%)
1.00	1.02 ± 0.26	11.32	2.41	11.04
2.50	2.67 ± 0.33	12.2	6.79	12.54
100.00	103.87 ± 5.77	6.23	3.32	9.35
800.00	809.29 ± 41.07	4.63	3.16	8.90

**Table 2:** Matrix effect and extraction recovery of compound **1** and psn357 in rat plasma (mean ± SD, *n* = 6).

Drugs	Conc. (ng/mL)	Matrix Effect		Extraction Recovery	
		Mean ± SD (%)	RSD (%)	Mean ± SD (%)	RSD (%)
Compound **1**	2.50	97.69 ± 9.36	9.58	91.55± 9.73	10.62
	100	96.52 ± 3.04	3.10	98.35 ± 6.42	6.36
	800	91.07± 6.05	7.12	97.54 ± 6.36	6.54
Psn357	100	91.09 ± 7.41	7.21	94.31 ± 6.29	6.15

**Table 3:** Stability of compound **1** under different storage conditions (*n* = 6).

Drug	Conc. (ng/mL)	Room Temperature (12 h)		Auto-Sampler (24 h)		Storage at −20 °C (10 days)		Thaw and Freeze (from −80 °C to 25 °C)	
		RE (%)	RSD (%)	RE (%)	RSD (%)	RE (%)	RSD (%)	RE (%)	RSD (%)
Compound **1**	2.5	0.50	8.37	13.81	12.24	12.30	13.74	12.40	13.60
	800	1.03	5.56	−3.20	7.71	−0.65	4.27	−3.64	7.31

**Table 4:** Dilution integrity of compound **1** in rat plasma (*n* = 6).

Working Concentration (ng/mL)	Dilution Factor	Concentration Measured (ng/mL)	Accuracy (%)	RSD (%)
1000	10	1051.9 ± 112.5	105.2	10.6
500	20	512.4 ± 33.6	102.5	6.6
100	100	99.5 ± 3.4	99.5	3.5

**Table 5:** Pharmacokinetic parameters of compound **1** in rats administered at low, medium and high concentrations (mean ± SD, *n* = 6).

Dosage (mg/kg)	AUC_0–t_ (h·μg/L)	AUC_0–∞_ (h·μg/L)	C_max_ (μg/L)	CL (L/h/kg)	T_1/2_ (h)	Vd (L/kg)
0.8 (iv)	894.91 ± 440.19	899.91 ± 441.17	1176.62 ± 361.31	1.00 ± 0.29	4.56 ± 2.17	6.43 ± 3.48
1.6 (iv)	1601.27 ± 372.00	1612.30 ± 371.05	2034.41 ± 289.32	1.04 ± 0.25	4.80 ± 3.22	7.51 ± 6.15
3.2 (iv)	3625.09 ± 969.80	3631.04 ± 972.98	3710.23 ± 682.91	0.94 ± 0.27	3.08 ± 0.58	4.14 ± 1.23

**Table 6:** Concentrations of compound **1** in plasma and brain tissue at different time points after administration to rats (mean ± SD, *n* = 6).

Conc. (ng/g)	0.25 (h)	2 (h)	8 (h)
C_Brain_	440.0 ± 19.1	111.0 ± 23.9	4.2 ± 7.2
C_Plasma_	4019.0 ± 677.0	1019.0 ± 275.0	32.9 ± 14.5

**Table 7:** C_Brain_/C_Plasma_ ratios of compound **1** in rats (mean ± SD, *n* = 6).

Ratio	0.25 (h)	2 (h)	8 (h)
C_Brain_/C_Plasma_	0.11 ± 0.019	0.11 ± 0.029	0.09 ± 0.148

**Table 8:** Pharmacokinetic parameters of compound **1** in plasma and brain tissue (*n* = 6).

Substrates	AUC_0–∞_ (h·ng/g)	C_max_ (ng/g)	T_max_ (h)	T_1/2_ (h)	MRT (h)
Plasma	6659	4889	0	1.14	1.47
Brain tissue	668	440	0.25	1.18	1.65

## Data Availability

The data presented in this study are available on request from the corresponding author.

## Sample Availability

Samples of the compounds are available from the corresponding author.
